# Representation Learning for Interpersonal and Multimodal Behavior Dynamics: A Multiview Extension of Latent Change Score Models

**DOI:** 10.1145/3577190.3614118

**Published:** 2023-10-09

**Authors:** Alexandria K. Vail, Jeffrey M. Girard, Lauren M. Bylsma, Jay Fournier, Holly A. Swartz, Jeffrey F. Cohn, Louis-Philippe Morency

**Affiliations:** Carnegie Mellon University, Pittsburgh, Pennsylvania, USA; University of Kansas, Lawrence, Kansas, USA; University of Pittsburgh, Pittsburgh, Pennsylvania, USA; The Ohio State University, Columbus, Ohio, USA; University of Pittsburgh, Pittsburgh, Pennsylvania, USA; University of Pittsburgh, Pittsburgh, Pennsylvania, USA; Carnegie Mellon University, Pittsburgh, Pennsylvania, USA

## Abstract

Characterizing the dynamics of behavior across multiple modalities and individuals is a vital component of computational behavior analysis. This is especially important in certain applications, such as psychotherapy, where individualized tracking of behavior patterns can provide valuable information about the patient’s mental state. Conventional methods that rely on aggregate statistics and correlational metrics may not always suffice, as they are often unable to capture causal relationships or evaluate the true probability of identified patterns. To address these challenges, we present a novel approach to learning multimodal and interpersonal representations of behavior dynamics during one-on-one interaction. Our approach is enabled by the introduction of a multiview extension of latent change score models, which facilitates the concurrent capture of both inter-modal and interpersonal behavior dynamics and the identification of directional relationships between them. A core advantage of our approach is its high level of interpretability while simultaneously achieving strong predictive performance. We evaluate our approach within the domain of therapist-client interactions, with the objective of gaining a deeper understanding about the collaborative relationship between the two, a crucial element of the therapeutic process. Our results demonstrate improved performance over conventional approaches that rely upon summary statistics or correlational metrics. Furthermore, since our multiview approach includes the explicit modeling of uncertainty, it naturally lends itself to integration with probabilistic classifiers, such as Gaussian process models. We demonstrate that this integration leads to even further improved performance, all the while maintaining highly interpretable qualities. Our analysis provides compelling motivation for further exploration of stochastic systems within computational models of behavior.

## 1 INTRODUCTION

To address mental health concerns successfully, it is critical to provide individuals with the necessary support to ensure their commitment to accomplishing their therapeutic treatment. One of the most important elements in fostering such commitment is the cultivation of a positive relationship between the client and the therapist. Empirical evidence has indicated that clients who share a positive relationship with their therapist are less likely to discontinue therapy [3] and more likely to experience favorable treatment outcomes [7, 36]. Therefore, it is essential to monitor the development of this relationship over the course of treatment to allow the therapist to adjust their approach to better meet the needs of the client. Unfortunately, obtaining genuine feedback from therapy clients can prove to be a challenge: clients often express hesitation due to concerns about confidentiality, fear of negative consequences, or a desire to please the therapist [37, 53]. However, computational modeling techniques have demonstrated considerable potential in simulating and forecasting other social constructs.

The task of modeling of human behavior is a challenging one, as it involves many factors. One such factor is the need to consider how each person affects and is affected by the other people around them [51, 60]. This reciprocity between interacting people is one of the greatest influences on an individual’s behavior in such contexts [31]. Furthermore, modeling social behavior during therapeutic treatment can be even more challenging than modeling social behavior in other contexts. Clients often exhibit greater vulnerability, openness, and self-reflection during therapy than they do in their everyday behavior [19]. This heightened state of engagement can lead to more intense emotional experience and expression, which can significantly affect the nature of the therapeutic conversation [35].

Another factor complicating the study of human behavior is the fact that information is communicated through many different modalities simultaneously. It is well-established that verbal and nonverbal behavior is interconnected [4, 17, 41] and offer different kinds of information [12, 13], but during therapy, the relationship between the two is particularly significant. Research has demonstrated that verbal behavior tends to more accurately reflect a person’s thoughts, while nonverbal behavior tends to more accurately reflect a person’s emotions [12, 45, 60]. However, this consistency (or inconsistency) of information provided across different modalities can reveal valuable insights into the client’s therapeutic experience [4, 29, 44].

Finally, when studying human behavior, it is imperative to acknowledge that our behavior is not static, but rather changes over time. Examining the dynamics of behavior is critical, as it allows for the identification of patterns and trends, and potentially even recognition of causal relationships between variables [8, 57]. Observing how an individual adapts their behavior in response to changes in others’ behavior can provide a wealth of information about the nature of their relationship [22, 34]. This observation is particularly valuable in the therapeutic context, where the client’s reactions to different prompts or actions of the therapist also serve as valuable indicators of their current mental state [8, 40].

This paper proposes a novel methodology for developing effective representations of human behavior during social interaction. Our suggested approach uses structural equation modeling to learn a representation of behavior dynamics that can offer a more comprehensive understanding of the causal relationships between behaviors and how each person’s behavior affects and is affected by the behavior of others. We demonstrate an application of this approach in evaluating the strength of the relationship between a client and therapist during therapy sessions, which can be a particularly challenging context. This methodology has the potential to provide new and valuable perspectives into behavior patterns across individuals, modalities, and time.

## 2 PROPOSED MODEL

Our approach to modeling behavior dynamics involves a three-step process. First, we introduce our novel multiview extension of latent change score models, which allows for the simultaneous capture of multimodal and interpersonal dynamics. We then demonstrate how these models are used to learn rich representations of behavior. Finally, we employ these representations as input for a predictive model, enabling us to make accurate predictions for practical implementation.

### 2.1 Multiview Latent Change Score Model

A well-defined structure is essential for accurate and reliable structural equation model-based analysis. In this study, we extend the structure of latent change score models, a family of models that are frequently used in psychological research for the study of longitudinal data [42]. In particular, we define a *multiview latent change score model* that allows us to simultaneously model patterns between modalities and individuals throughout an interaction.

At the highest level, latent change score models are SEM structures that aim to estimate changes in a given variable over time. These models attempt to identify the underlying structure of these changes through the use of both observed variables and latent factors. From a machine learning perspective, these models resemble an approach that takes advantage of supervised and unsupervised techniques to analyze longitudinal data. By incorporating domain-informed hypotheses about unobserved confounding factors (i.e., latent factors), these models help us better understand the relationship between variables.

**Figure 2:**
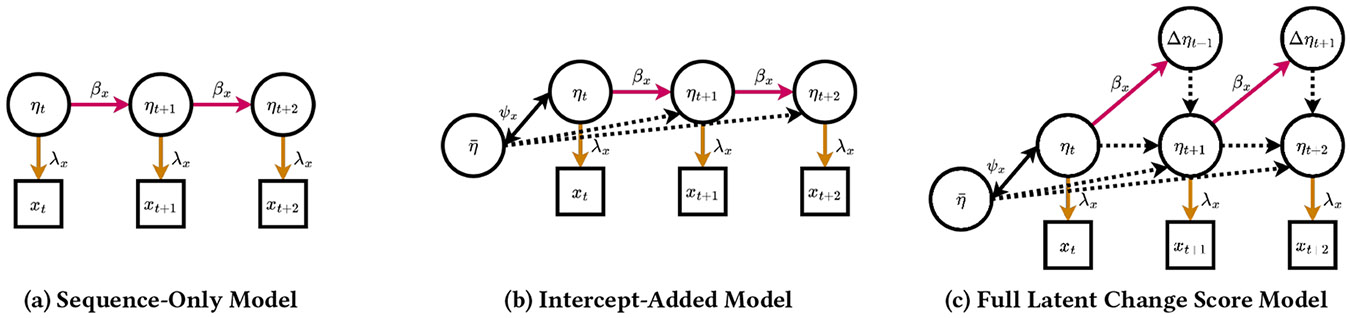
Ablation steps to build the univariate latent change score model. Colored paths represent paths tied to each other (with the exception of black paths). Note that for clarity, self-variances are excluded from the illustration. Dotted lines indicate parameters constrained to the unit weight.

The standard single-view latent change score model is illustrated in Figure 2c. Although the latent change score model can contain any number of measured time points (greater than two), the number of points to include is highly dependent on the available data [24, 42]. In our case, we have a few different elements to consider.

- We need our chosen duration of $x_{t}$ to be a reliable measure of behavior during that time interval, e.g., to ensure that both individuals have sufficient time for speaking and listening behavior during each segment.
- Based on our duration of $x_{t}$, we need to ensure that the duration of each complete sequence (duration$(x_{t})\times k$ points) allows a sufficient number of sample sequences to be drawn from the entire session to perform meaningful statistical modeling.
- We must ensure that we have enough time points per sequence to accurately estimate the free parameters in the model.

To achieve these objectives, it is crucial to select an appropriate duration and quantity of $x_{t}$ that balances the need for an accurate representation of individual behaviors with the need to maintain a suitable number of sample sequences and data points for robust statistical analysis. We selected a 45-second window for each time point $x_{t}$ after evaluating the fit of the single view model on each of our behavior markers. Given this 45-second window, our average session duration of 50–60 minutes, and our models as specified earlier, we decided to proceed with a three-point sequence (see subsection 3.1, Data Set). This decision results in having 60–80 input sequences per model, which is consistent with the typical suggestion of 10–20 sequences per free parameter [24]. Therefore, the single-view model upon which we expand our analysis consists of a sequence of three observed variables and five latent factors. We deconstruct this model into three ablation phases to define and later demonstrate the significance of each component (see Figure 2 for an illustration of each step).

**Step 1: Latent sequence** (Figure 2a). The core of this model is the representation of longitudinal data in its most primitive stage. The basic implementation of a three-part sequential SEM consists of the three measured variables ($x_{t}$, $x_{t+1}$, $x_{t+2}$) loaded onto their respective latent factors ($\eta_{t}$, $\eta_{t+1}$, $\eta_{t+2}$). These loadings ($\lambda_{x}$) represent the degree to which the latent construct explains the variance of the measured variable. This connection encodes the hypothesis that each measurement is the sum of the “true” latent value plus some amount of measurement error (self-variance, $\theta_{x}$). We constrain these loadings to be equivalent for each time point because we expect that this relationship will not change over time, and doing so will improve the estimation and interpretability of the model. The three latent factors are connected with one-way causal paths, suggesting that the value at each time point is influenced by the value at the previous time point, along with the variance of the latent factor itself ($\psi_{xt}$). At this point, we can define our model using the following equations.


(1) $$x_{t}=\lambda_{x}\eta_{t}+\theta_{x}$$

(2) $$x_{t+1}=\lambda_{x}\eta_{t+1}+\theta_{x}\;\;\;\;\;\eta_{t+1}=\beta_{x}\eta_{t}+\psi_{xt}$$

(3) $$x_{t+2}=\lambda_{x}\eta_{t+2}+\theta_{x}\;\;\;\;\;\eta_{t+2}=\beta_{x}\eta_{t+1}+\psi_{xt}$$

**Step 2: Intercept** (Figure 2b). The next component that we add to the model is the sequence *intercept* ($\bar{\eta }$). This intercept represents the value of a construct at the first time point, serving as a baseline against which future values of the construct are compared. Neglecting to include an intercept would represent the assumption that all sequences begin at the same value: an untenable premise. We can now define our latent factors with the following equations; note that the measured variables ($x_{t}$, $x_{t+1}$, $x_{t+2}$) will retain the same definition throughout.


(4) $$\eta_{t}=\bar{\eta }+\psi_{xt}$$

(5) $$\eta_{t+1}=\beta_{x}\eta_{t}+\bar{\eta }+\psi_{xt}$$

(6) $$\eta_{t+2}=\beta_{x}\eta_{t+1}+\bar{\eta }+\psi_{xt}$$

**Step 3: Latent change factors** (Figure 2c). A defining element of the latent change score model is the inclusion of *latent change factors* ($\Delta \eta_{t+1}$, $\Delta \eta_{t+2}$). These second-order latent factors represent the change in the first-order latent factors over time. Inclusion of these factors helps the model account for variability in the dynamics across individuals — or, in our case, across different moments in the therapy session.


(7) $$\eta_{t}=\bar{\eta }+\psi_{xt}\;\;\;\;\;\;\;\;\;\bar{\eta }=\psi_{x}\eta_{t}$$

(8) $$\eta_{t+1}=\bar{\eta }+\eta_{t}+\Delta \eta_{t+1}+\psi_{xt}\;\;\;\Delta \eta_{t+1}=\beta_{x}\eta_{t}+\psi_{\Delta xt}$$

(9) $$\eta_{t+2}=\bar{\eta }+\eta_{t+1}+\Delta \eta_{t+2}+\psi_{xt}\;\;\Delta \eta_{t+2}=\beta_{x}\eta_{t+1}+\psi_{\Delta xt}$$

**Figure 3:**
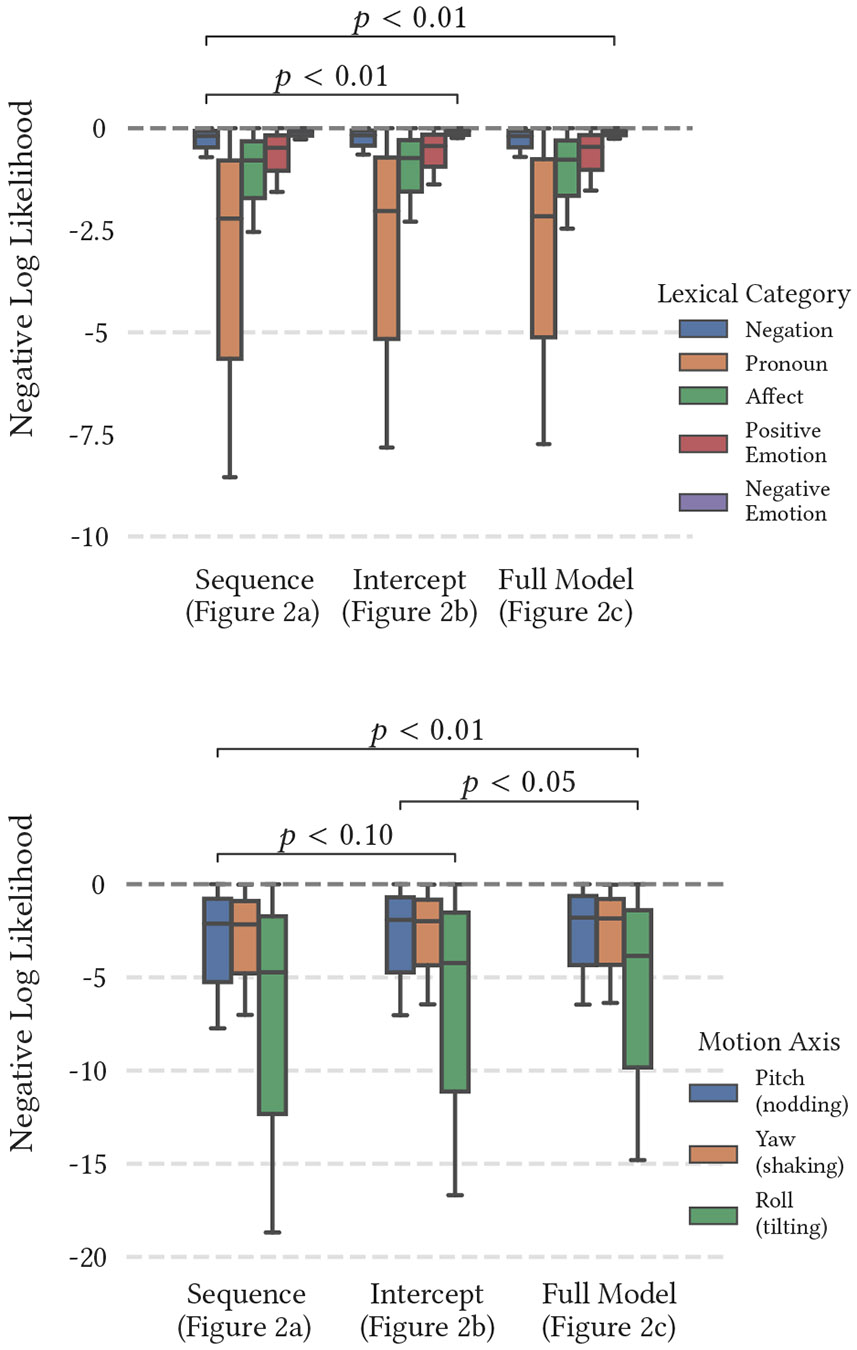
Average negative log-likelihood of converged unimodal models across behavioral markers, with statistically significant differences annotated. Ablation across the sequence-only, added-intercept, and full variants of the latent change score model (LCSM; Figure 2) suggests that the inclusion of each additional structural element improves the fit of the model. Note that head motion-based models exhibit a significantly poorer fit in a unimodal context compared to language-based models.

Figure 3 illustrates the fit achieved by the unimodal version of the model. In the case of this analysis, our objective is to simulate behavior dynamics between modalities and individuals during the interaction of a therapist and their client. Therefore, we extend the standard latent change score model by creating a multiview extension to incorporate multiple modalities and individuals in the analysis.

**Figure 4:**
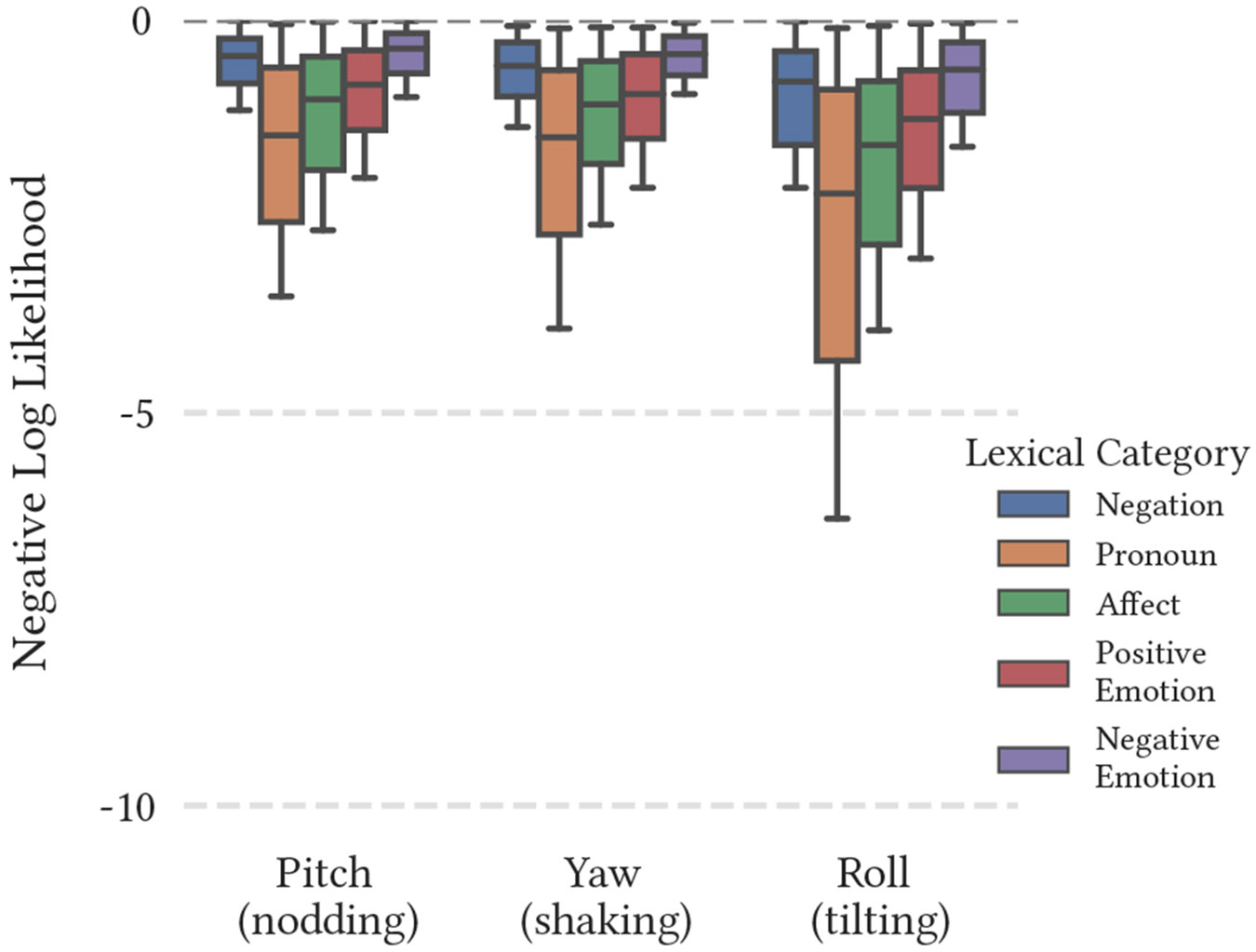
Average negative log-likelihood of converged bimodal models. Each model was trained upon multiple modalities within the same individual.

**Figure 5:**
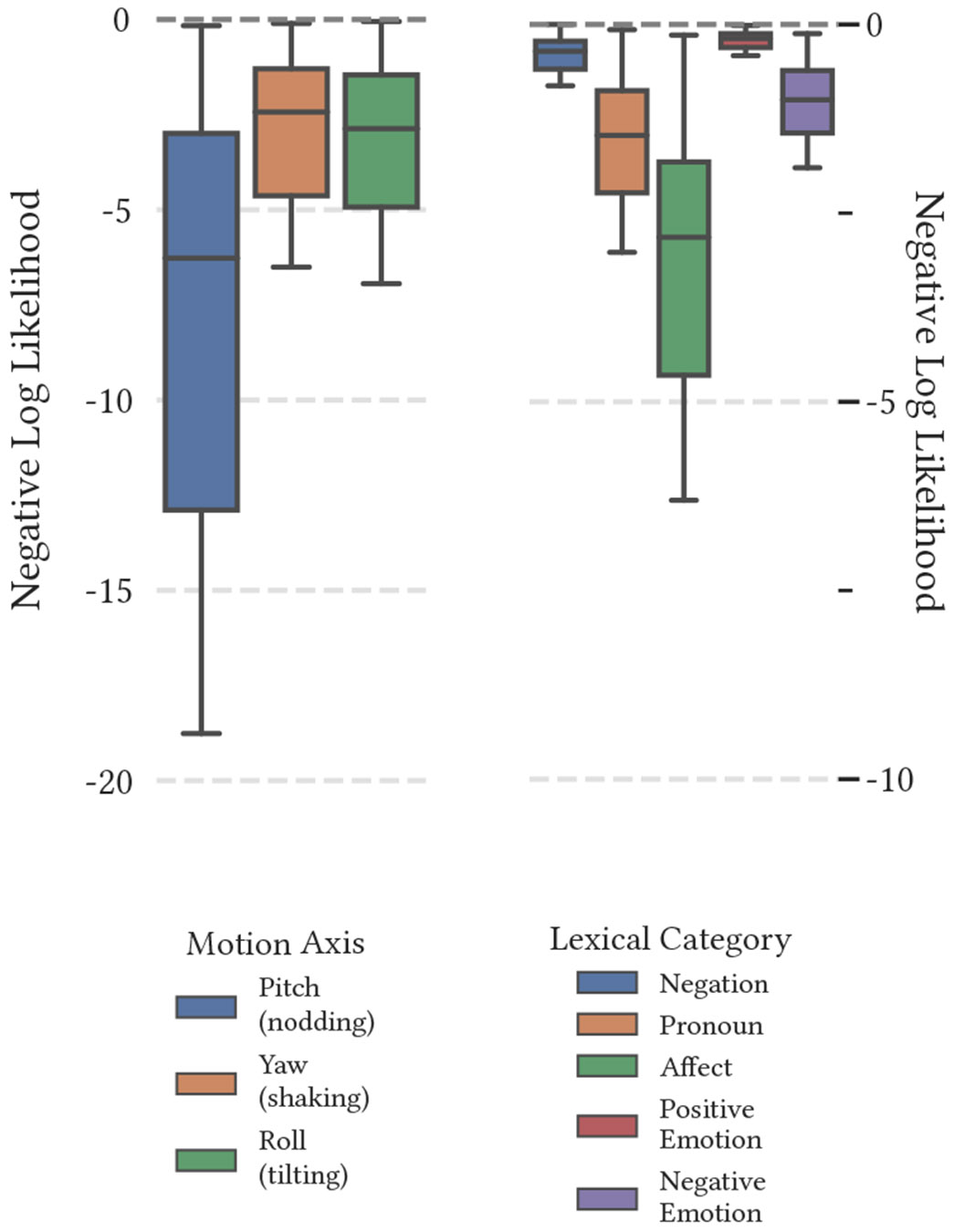
Average negative log-likelihood of converged dyadic models. Each model was trained upon identical features across both client and therapist.

**Step 4: Multiview extension.** The bivariate extension of the latent change score model enables the study of two forms of behavior dynamics over time, as well as cross-modal (Figure 4) or cross-individual (Figure 5) interactions between these temporal dynamics. For example, if the client starts nodding more frequently than before, does the therapist also begin nodding more than before? Is the client’s head motion related to the discussion about emotions? Inclusion of covariance parameters across latent constructs, intercepts, and change factors of different behaviors enables a deeper investigation of these research questions.

Ultimately, however, our goal is to model the details of the temporal behavior dynamics between modalities *and* individuals. To achieve this, we further extend the bivariate latent change score model to construct a *multiview latent change score model*. By integrating cross-modal interactions and individual differences, this multiview extension offers valuable insight into the intricate patterns of therapist-client interactions, facilitating a more nuanced understanding of the factors influencing therapy outcomes.

Structural equation modeling (SEM) is a multivariate statistical approach used to analyze complex relationships between latent and observed variables [16, 38]. Generally confirmatory in nature, SEM aims to test whether a hypothesized model fits a given dataset, involving the use of several mathematical *equations* describing a hypothesized *structure* of the data. This structure defines a set of relationships between latent and observed variables, such as factor loadings, causal pathways, and covariance matrices. If the model fits the data well, its structure provides us with insight into the underlying driving behavior patterns in the data, while also taking into account measurement errors and potentially confounding factors. In general, SEM has become increasingly popular for interdisciplinary research due to its ability to capture complexity within systems without sacrificing interpretability [49, 56, 62].

We selected this modeling technique over other traditional machine learning models for several reasons. The primary advantage we value is the interpretability of SEM, which provides more understandable and approachable explanations of the relationships between variables. SEM provides a graphical representation of the model that helps visualize complex relationships between factors. Furthermore, many popular black-box frameworks used in machine learning, such as deep neural networks, require large amounts of training data before producing meaningful results. In contrast, SEM can provide insight from smaller sample sizes with fewer observations since it combines data-driven parameter training with expert domain knowledge [33, 49]. This benefit is even more advantageous to our domain than most areas of multimodal research: the additional overhead and sensitivity required to collect rich multimodal behavior data, especially in healthcare, often leads to a smaller number of available observations than is available for other research areas.

### 2.2 Representation Learning

The ultimate objective of the SEM framework is to minimize the difference between the covariance matrix observed in the data and the covariance matrix implied by the model. Consequently, the appropriate approximation of the covariance matrix is of vital importance for our analysis. We note that the standard calculation of the covariance matrix is suboptimal for our use case: we cannot assume that our data are normally distributed (we would expect a long-tailed distribution), nor does our dataset contain an overly large number of observations (conventional wisdom suggests that the standard calculation requires 10–20× observations as the number of observed variables; [25, 39]). For these reasons, we turn to the asymptotic distribution-free covariance estimation method.

The asymptotic distribution-free covariance matrix is calculated using Spearman’s rank coefficient, a nonparametric measure of correlation based on the order of values [63], in contrast to the standard calculation which uses the normality-assuming Pearson’s coefficient based on the raw values [46]. The method of asymptotic distribution-free covariance estimation has also been shown to improve the performance of covariance-based models when an analysis is limited by smaller data sets [43].

Our goal is to minimize the difference between this sample covariance matrix and the model-implied covariance matrix. The model-implied covariance matrix is calculated with

(10) $$\Sigma_{M}=\Lambda {\left(I-B_{0}\right)}^{-1}\Psi {\left({\left(I-B_{0}\right)}^{-1}\right)}^{T}\Lambda^{T}+\Theta ,$$

where Λ, Θ, Ψ, and $B_{0}$ are the four parameter matrices that specify the model^1^.

For an SEM with $n_{m}$ measured variables and $n_{l}$ latent factors, these matrices are

- Factor loadings (Λ), the regression coefficients of unobserved latent factors on observed measured variables, of shape $n_{m}\times n_{l}$;
- Residual variances of observed variables (Θ), including measurement error, of shape $n_{m}\times n_{m}$;
- Variances and covariances of latent variables (Ψ), of shape $n_{l}\times n_{l}$; and
- Causal pathways (B), representing causal relationships between latent variables, of shape $n_{l}\times n_{l}$.

The models were trained using the Adam optimization algorithm [32] with an initial learning rate of 0.01 and the weighted squared error loss function as the minimization objective. We selected the weighted squared error loss function because, unlike other common SEM loss functions, such as maximum likelihood, the squared error loss does not assume any normality of the data [33].


(11) $$\text{loss}={\left(\Sigma_{S}-\Sigma_{M}\right)}^{T}W\cdot (\Sigma_{S}-\Sigma_{M})$$

In this case, the weight matrix is set to the inverse of the covariance matrix of the sample data ($W=\Sigma_{S}^{-1}$). Using these weights is one way to place more emphasis on data with a smaller variance and less emphasis on data with a larger variance, to reduce the impact of observations with larger errors or greater uncertainty [20].

The training procedure was repeated multiple times with random initialization. In addition to improving the robustness of the model, drawing more samples from the distribution of parameter estimates helps us to define a prior distribution for the second phase of our analysis (see section 3, Experimental Setup). By approximating a range of values rather than a singular value, we can preserve data regarding the uncertainty of our parameter estimates. Retaining this uncertainty allows us to make more informed interpretations of the predictive model.

### 2.3 Gaussian Process Regression

For our study, we have emphasized the Gaussian process (GP) regressor as our preferred predictor. It is relevant to note that, despite the ‘Gaussian’ name, GPs are not limited to modeling data believed to be drawn from an underlying Gaussian distribution. Instead, the name is derived from the fact that GPs learn each parameter estimate as a Gaussian distribution [50]. This is in contrast to various contemporary machine learning models that typically approximate parameter estimates as fixed or point values. Incorporating uncertainty into a model, similar to the benefits of structural equation modeling, can improve the robustness of the model when dealing with real-world data, which are often affected by measurement error and other noise. Furthermore, Gaussian process models possess the capability to effectively approximate nonlinear associations, as they are based on kernel functions. This attribute differentiates them from other probability-based regression models, such as Bayesian regression, which are based on linear functions [18].

## 3 EXPERIMENTAL SETUP

In addition to simply applying structural equation models to our data, we also aim to demonstrate the practicality of these features. This is achieved by presenting a comparison of various predictive models that utilize said features to forecast the working alliance ratings of both therapist and client. The data used for this analysis is derived from the behavior of therapists and clients during therapy sessions, specifically their head motion and language features. Our ultimate objective is to use these markers to construct interpretable predictive models that enable us to gain a more nuanced understanding of these underlying behavior dynamics. The results illustrate the utility of structural equation modeling as a form of representation learning for systems of behavior.

### 3.1 Data Set

The audiovisual recordings used in this analysis include 266 therapy sessions, with the participation of 39 unique clients and 11 unique therapists[58]. Each therapist worked with 3 to 5 different clients, each client attending 6 to 8 sessions that lasted between 40 and 60 minutes. Therapy sessions were held in a private setting and recorded with the consent of the clients and the therapists.

Participants were recruited from a research registry, printed material advertising the study personal referrals. For inclusion in the study, participants were required to be between 18 and 65 years of age, meet the diagnostic standards for major depressive disorder according to DSM-5 [2], experience moderate or greater depressive symptoms (as indicated by a Hamilton Rating Scale for Depression score of 14 or higher; [26]), and be able and willing to provide informed consent. Individuals with comorbid psychotic disorders, active suicidal or homicidal ideation, chronic depressive symptoms, or current misuse of substances or alcohol were excluded from the study. Participants with suspected psychosis or active suicidal ideation with intent or a plan to harm themselves were referred to the psychiatric emergency room.

#### 3.1.1 Feature Extraction: Head Motion.

Head motion features were extracted from patient and clinician videos using OpenFace [9]. The extracted features represented the total degree of head motion in radians for each axis (pitch, yaw, and roll) within that time window. Data were grouped by a window size of 45 seconds, which was selected to guarantee a sufficient number of data points per session to attain acceptable statistical power in later analysis (see subsection 2.3; Gaussian Process Regression).

To reduce tracking noise, two measures were implemented. First, frames that had a confidence level lower than 90% were eliminated^2^ and linear interpolation was applied to fill the gaps, which was considered satisfactory given that the data were collected at a consistent rate. To further reduce tracking noise, a Savitzky-Golay filter was utilized to smooth the data, as it is recognized to be more effective than a moving average filter in maintaining the original shape of the data given its polynomial fitting [55]. Implementing these measures ensured a cleaner and more reliable data set for analysis.

#### 3.1.2 Feature Extraction: Language Use.

The audio recordings of the sessions were transcribed using a machine transcription service [59]. From these transcripts, we extracted various lexical categories using the LIWC tool (Linguistic Inquiry and Word Count; [47]), which has shown validity in measuring verbal dialogue and language usage in multiple domains [15, 48, 54]. For this study, we focus on the use of five particular lexical categories of language:

- *negations*, such as “no”, “never”, and “not”;
- *pronouns*, such as “I”, “them”, and “itself”;
- *affective words*, such as “nervous”, “ugly”, and “bitter”;
- *positive emotions*^3^, such as “happy”, “pretty”, and “good”; and
- *negative emotions*^3^, such as “hate”, “worthless”, and “enemy”.

Existing literature has shown that these specific linguistic categories are strong indicators of both an individual’s mental well-being and interpersonal connections. Previous research has shown that overuse of negative words can cause increased tension between speakers [61]. However, negations can also be used to soften potentially adversarial or distressing statements during difficult conversations to preserve rapport [11]. The use of pronouns and positive emotion words tends to improve the listener’s perception of empathy, trust, or closeness [1, 23]. Negative emotion words can serve a similar purpose as negations: while often linked to social tension or negative communication spiraling at a broad level [5, 21], negative emotion words can also facilitate collaborative problem solving and understanding when communicated with respect and empathy [52].

#### 3.1.3 Target Variable: Working Alliance Ratings.

The working alliance in therapy refers to the collaborative relationship that develops between a therapist and the client throughout treatment and the degree to which they work together effectively [10]. A strong working alliance fosters trust and open communication between the client and the therapist, which is known to contribute to better therapeutic outcomes [29]. After the end of each therapy session, both the therapist and the client participants completed the therapist and client versions of the short form of the Working Alliance Inventory (WAI-SR; [28, 30]), a widely used measure of alliance in therapy. The WAI consists of three subscales that measure the three distinct components of a working alliance:

- the *goal* subscale, which evaluates the individual’s belief that participants agree on the overall objectives of the treatment;
- the *task* subscale, which evaluates the individual’s belief that participants agree on the steps required to achieve those goals; and
- the *bond* subscale, which evaluates the individual’s emotional respect and trust for the other participant.

Each subscale consists of statements that the individual rates on a five-point Likert-type scale ranging from “seldom true” to “always true”. The client version of the inventory contains 12 items, while the therapist version contains 10 items. For the purposes of this analysis, we combine the *task* and *goal* subscales due to very high correlation: these two subscales achieve Pearson’s correlation coefficient of r=0.96 between them.^4^ Representative items for each subscale are presented in Table 1.

### 3.2 Baseline Models

We select a small set of popular machine learning models to compare: ElasticNet, support vector regression, random forests, and the Gaussian process regressor. We selected these algorithms for their ability to perform well on small data sets. We have particular interest in the Gaussian process regressor because it can incorporate the information about uncertainty in the parameter estimates from the structural equation model. We also compare our multiview LCSM-based feature set against other frequently-used sets of sequence features: aggregate features (entropy, mean changes, variance, etc. [14]), cross-correlation features, and the combination of aggregate and cross-correlation features.

Model hyperparameters were automatically selected using a leave-one-therapist-out approach to reduce the risk of train-test data contamination. In this approach, each therapist (n=11) acted once as the test set: all sessions conducted by that therapist were designated as the test set, while all other sessions were allocated to the training set. Validation for each fold was conducted in a similar manner within the training set, with one therapist’s sessions being used for validation and the remaining sessions used for training. Features were recalculated with every training run to prevent dependence on values from the test set. Prediction performance was measured using the root mean squared error (RMSE) metric. One advantage of RMSE over some comparable metrics, such as the coefficient of determination $R^{2}$, is that it is defined in the same units as the output variable — in this case, working alliance ratings — and its stability in smaller data sets.

Table 2 presents a comparison of the test-set performance for each prediction model. Results demonstrate that the multiview LCSM features perform at the same level or surpass other commonly-used feature sets for temporal behavior analysis.

### 3.3 Behavioral Dynamics Features

The objective of our predictive models is to determine the extent to which the structure of the multimodal behavior dynamics during a therapy session can provide information on the strength of the overall working alliance shared by the client and the therapist. To achieve this, we propose a novel approach that incorporates the parameters of the structural equation models, namely Λ, Ψ, Θ, and B, which were introduced and estimated in subsection 2.2, Representation Learning. These parameters serve as a collection of computational metrics that allow us to quantify the behavior dynamics observed throughout each session. However, translating parameter estimates from one model into input features for another model presents an additional challenge, as the uncertainty information provided by the initial model estimates is lost. Therefore, we must take some additional steps to integrate this uncertainty information obtained from the initial model estimates into the subsequent model.

In structural equation modeling, each parameter estimate is represented as a distribution that includes a central value and an estimation of the standard error. This standard error serves to measure the accuracy of the parameter estimate and to indicate the degree of variability from the potential actual parameter value. Through multiple initializations of the models trained in section 2 (Proposed Model), a set of samples has been produced from the distribution of possible true parameter values, each with its own corresponding measure of confidence. The majority of models we present cannot take advantage of this data: however, the improved performance when it is provided to the Gaussian process regressor demonstrates its value (see Table 2).

## 4 RESULTS AND DISCUSSION

Our objective was to demonstrate the use of structural equation modeling as a means of representation learning for machine learning models. Our findings indicate that the models display a reasonable fit, the features constitute valuable information for prediction tasks, and we are now able to showcase the potential for interpretation that this approach offers.

Table 3 presents the top three features, ranked by weight, for each of the target labels (task+goal ratings and bond ratings, each for both client and therapist). Some of the significant features are as expected, while others are not. For instance, we can observe that the client’s overall use of negative emotion words (the intercept; Table 3c) is positively associated with the client’s bond rating. This could be due to the fact that clients who are more willing to express their negative emotions to the therapist may feel a stronger connection with them, or that clients who feel more connected to the therapist may be more willing to share their negative emotions [6]. Additionally, we observe a that a stronger covariance between the use of pronouns by the therapist and the client’s nodding (Table 3b) is linked to higher ratings of task and goal by the therapist. Pronoun words, such as “I, you, they”, may indicate that when the therapist is discussing the client (“you”) and the client nods, the therapist interprets this as a sign of agreement. However, we also note some unforeseen relationships. For instance, the covariance between the client’s nodding and the client’s use of negative emotion words is inversely related to the client’s assessment of the task and goal. Future work is necessary to determine the underlying reasons for this, but it is noteworthy to observe.

## 5 CONCLUSION

We have presented a novel methodology for developing computational representations of behavior that integrate information from multiple modalities, individuals, and time points. Our technique builds upon an existing structural equation modeling framework. Specifically, we define a multiview extension of the latent change score model. Our analysis indicates that this structure does fit data well in our use case, suggesting that it is indeed finding patterns in the data. We use the learned parameters of this model as input features for a secondary, predictive model, and demonstrate that the performance achieved using these features is comparable to that achieved using than many classic features for this task. Our findings demonstrate that learning features through this particular form of model training yields rich information about specific areas of uncertainty, and that integration of this knowledge into models that are equipped to handle such information improves performance further. This approach to learning representations of multimodal, interpersonal, and temporal behavior creates novel opportunities for learning about and simulating human behavior.

## Figures and Tables

**Figure 1:**
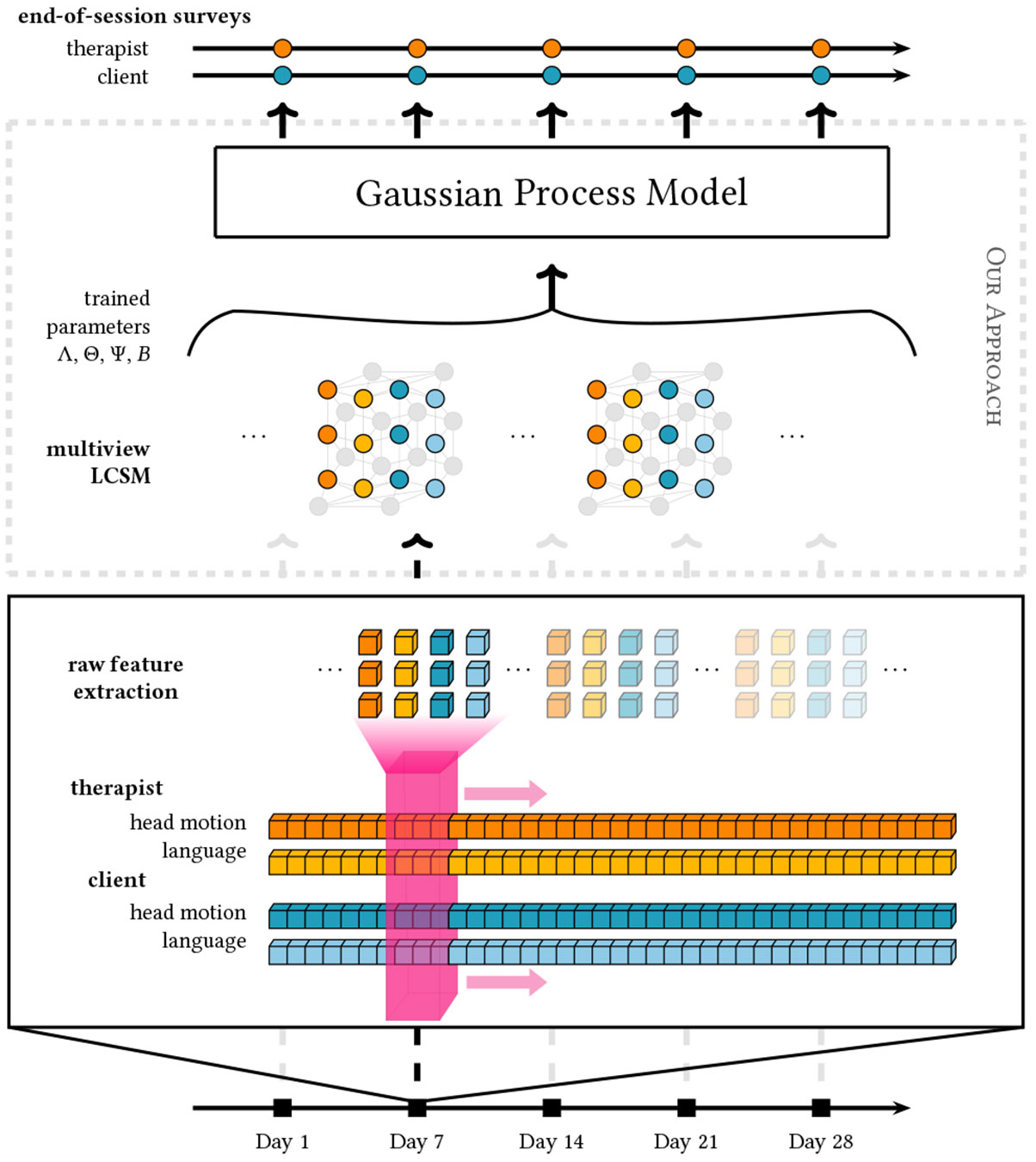
An overview illustration of the methodology presented in this work.

**Table 1:** Sample items from both therapist and client versions of the Working Alliance Inventory.

Goal Subscale	Task Subscale	Bond Subscale
[Therapist] and I collaborate on setting goals for my therapy. [Therapist] and I have established a good understanding of the kind of changes that would be good for me. We are working towards mutually agreed upon goals. [Client] and I have a common perception of his/her goals.	What I am doing in therapy gives me new ways of looking at my problem. [Therapist] and I agree on what is important for me to work on. [Client] and I agree about the steps to be taken to improve his/her situation. [Client] and I both feel confident about the usefulness of our current activity in therapy.	I believe [Therapist] likes me. I feel that [Therapist] appreciates me. I feel [Therapist] cares about me even when I do things that he/she does not approve of. I appreciate [Client] as a person. [Client] and I respect each other.

**Table 2:** Performance metrics of predictive models: Root Mean Squared Error (mean and standard deviation). Each model was trained and tested with each of the feature sets of interest: aggregate statistics, cross-correlation statistics, combination of aggregate and cross-correlation statistics, and our multiview LCSM-based features without uncertainty information. For comparison, we also include the performance of the Gaussian Process model when it is provided with the uncertainty information from the multiview LCSM.

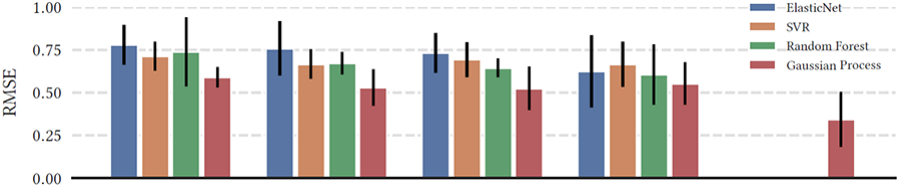					
	Aggregate	Cross-Correlation	Agg. + Cross-Corr.	Multiview LCSM	Multiview LCSM w/Uncertainty
ElasticNet	0.7791 (0.2294)	0.7588 (0.3164)	0.7320 (0.2308)	**0.6255 (0.4209)**	–
SVM	0.7131 (0.1696)	0.6661 (0.1699)	0.6935 (0.2026)	**0.6653 (0.2637)**	–
Random Forest	0.7383 (0.4028)	0.6719 (0.1320)	0.6450 (0.1056)	**0.6056 (0.3484)**	–
Gaussian Process	0.5909 (0.1174)	0.5298 (0.2129)	0.5245 (0.2536)	0.5534 (0.2465)	**0.3426 (0.3193)**

**Table 3:** Top three features in the Gaussian process model by average weight for each of the target labels.

LCSM Parameter	Weight
**Covariance:** client pitch motion (nodding) & client negative emotion words	−1.3021
**Transition:** client pitch motion (nodding) over time	1.0398
**Covariance:** therapist pronoun words & client pronoun words	0.9964
(a) Client task + goal ratings.	
LCSM Parameter	Weight
**Intercept:** client pronoun words	1.9289
**Covariance:** therapist pronoun words & client pitch motion (nodding)	0.9715
**Intercept:** therapist pronoun words	0.8118
(b) Therapist task + goal ratings.	
LCSM Parameter	Weight
**Intercept:** client negative emotion words	1.7728
**Covariance:** therapist pronoun words & client pronoun words	1.2825
**Covariance:** therapist affective words & client yaw motion (shaking)	−1.0237
(c) Client bond ratings.	
LCSM Parameter	Weight
**Covariance:** client roll motion (tilting) & client affective words	1.3413
**Intercept:** client yaw motion (shaking)	1.1930
**Covariance:** therapist affective words & client negative emotion words	1.0697
(d) Therapist bond ratings.	

## Footnotes

1 Although much of the statistical literature presents SEM analyses in the fully-specified “LISREL” notation convention, we present our model in the abbreviated “all-y” convention for simplicity and accessibility (see [27] for more information).

2 Approximately 6% of video frames were excluded for low tracking confidence.

3 Note that *positive emotion words* and *negative emotion words* are subcategories of *affective words*.

4 For comparison, the correlation between the bond subscale and each of these factors is r=0.51 and r=0.52, respectively.
